# Assessment of Tree Leaves Flakes Mixed with Crude Glycerol as a Bioenergy Source

**DOI:** 10.1155/2016/5805806

**Published:** 2016-06-19

**Authors:** Ali Hilal-AlNaqbi, Salah B. Al-Omari, Mohamed Y. E. Selim

**Affiliations:** Department of Mechanical Engineering, College of Engineering, UAE University, Al Ain, UAE

## Abstract

The gasification and combustion of dry tree leaves and the cogasification of dry tree leaves soaking crude glycerol were studied experimentally. An updraft fixed bed gasification and combustion system was built. The operation was conducted at different air to fuel ratios. Results show more stable combustion and more effective heat transfer to furnace walls for the cases when tree leaves flakes are mixed with 20 percent (on mass basis) of crude glycerol, as compared with the case when only dry tree leaves are used as fuel. TGA analysis was also conducted for the two fuels used under both air and nitrogen environments. For the crude glycerol, four phases of pyrolysis and gasification were noticed under either of the two surrounding gaseous media (air or nitrogen). For the dry tree leaves, the pyrolysis under nitrogen shows only a simple smooth pyrolysis and gasification curve without showing the different distinct phases that were otherwise identified when the pyrolysis is conducted under air environment. Moreover, the air TGA results lead to more gasification due to the char oxidation at high temperatures. DTG results are also presented and discussed.

## 1. Introduction

The forecast for energy use worldwide continues to show rising levels of demand over the next three decades, led by strong increases in most countries. A total world energy consumption is expected to rise from 2012 to 2040, by about 48% [1]. The growth in energy demand is a strong indicator of the economic growth [2, 3]. The strong projected economic growth rates in return drive the fast-paced growth in future energy consumption and hence justify the search for further renewable sources and the development of ways and designs for their effective use [4]. Although liquid fuels—mostly petroleum-based—remain the largest source of energy worldwide, natural gas is expected to become the fastest-growing fossil fuel [5], while coal remains, at least currently, the world's slowest-growing energy source [6].

CO_2_ emissions resulting from the combustion of the above conventional energy sources are expected to increase in the coming decades [7]. On the other hand, it has been proved that the use of combustible renewable biomass materials (e.g., those derived from nonedible vegetables), by virtue of the nature of their combustion characteristics, would lead to reduction in CO_2_ emissions [8, 9]. Growing more plants would therefore lead to multiple benefits, for example, the production of more biofuels that are associated with reduction in the emission of CO_2_, in addition to other beneficial environmental end effects such as attaining more green land and cleaner atmosphere. Renewable energy sources in general are the world's fastest-growing energy source and they are expected to continue being so over the coming decades [10]. Examples of these renewable energy sources include biofuels, solar and wind energies, and the energy of the tide waves [11–15]. The use of such renewable energy sources proved to be effective in the three main areas of energy utilization, namely, in power generation, air conditioning, and transportation [16].

Bioenergy sources include solids, liquids, or gases derived from vegetables or any wastes of biological origin, collectively named biomass. Examples of these biomass materials are many household wastes, industrial wastes, crops, and agricultural wastes [17–19]. Biomass fuels can either be used in direct combustion to produce heat or indirectly by converting them via different physical and chemical processes to various forms of useful higher level biofuels (see, e.g., [20–25]). Wood remains the largest biomass energy source today. This includes forest residues, such as dead trees, branches and tree stumps, wood chips, and even much of the municipal solid wastes. In this paper, we consider a common biomass material, namely, tree leaves that is abundant everywhere in the world and hence is believed to increasingly have a strong share in the global energy use. We hereby shed light on this special kind of biomass and its gasification and combustion characteristics.

Dry green tree leaves are a renewable fuel with reasonable price which can be stored for use. Moreover, minimum capital is required for energy extraction from this fuel. Generally, dry green tree leaves can be made available with present manpower and material sources in all countries; therefore electrical energy can be generated on a large scale at very low cost. From an environmental aspect, during burning of dry green tree leaves, the oxygen from the atmosphere combines with the carbon in the waste to produce CO_2_ and water. This CO_2_ and water are again available for other tree growth and hence the cyclic process continues making dry green tree leaves a renewable source of energy which does not contribute to global warming. Furthermore, low levels of sulfur and ash in dry green tree leaves combustion products prevent acid rain formation.

**Figure 1:**
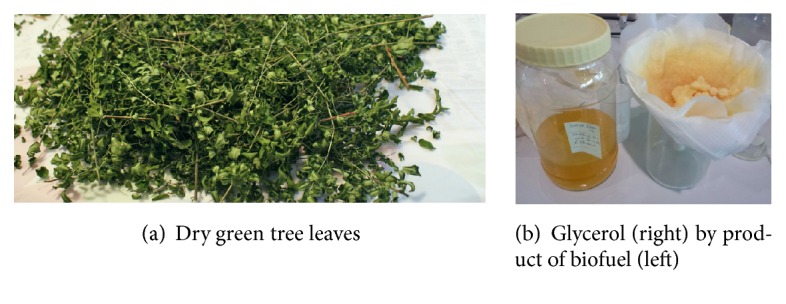
Fuels used.

The main objective of this work is to investigate the gasification and combustion in an updraft fixed bed of the dry green tree leaves on one hand and when the tree leaves flakes are wetted with crude glycerol on the other hand. Both of the tree leaves and the crude glycerol are considered as a waste material and they will be used here as biomass source to produce thermal energy. The dry green tree leaves used in this study are collected from local mixed trees as collected by local waste company. The crude glycerol used is the byproduct of the transesterification process to produce biodiesel fuel. Photos for the used dry green tree leaves and the crude glycerol are given in Figure 1.

The direct use of crude glycerol in gasification and combustion system proves to be a convenient and economical way to get rid in a useful manner of abundant amounts of waste material that increasingly is expected to become a burden on biodiesel production industry [26]. The treatment and purification of crude glycerol wastes to be used in subsequent industries (e.g., pharmaceutical industry) and or to be utilized as food supplement for animals are still at infancy stage and are not cost-effective enough to encourage their implementation at a wide scale, especially for small biodiesel production plants (see, e.g., [26, 27]). At the same time, selling crude glycerol as is at low prices to other industries while having the option to utilize it in-site for upper grade gases fuels production for power generation purposes seems not to be the optimal solution for the management of this kind of waste.

From the above, gasification of crude glycerol to produce high grade gaseous fuels that can be burnt efficiently and with reduced negative impact on the environment looks to be the optimal approach and hence different researches considered the optimization of such processes in different ways (see, e.g., [26–29] and the references cited therein).

**Figure 2:**
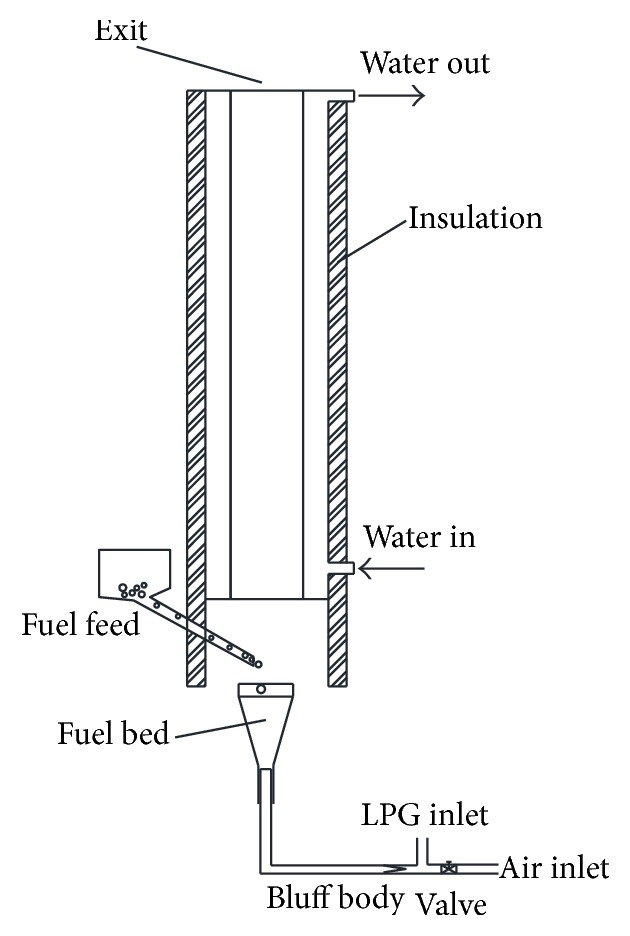
Schematic diagram of the used test rig.

This study considers the cogasification and combustion of two kinds of waste, namely, the dry green tree leaves and the leaves mixed with crude glycerol. An updraft fixed bed gasifier attached to a cylindrical furnace equipped with a water jacket to quantify heat transfer was designed and built for that purpose (see Figure 2). The study considers the gasification of these fuels in the bed at different temperatures and different air flow conditions. The crude glycerol in the used waste-material mix constitutes 25 percent on mass basis.

The final exhaust gases are collected at the exit of the furnace as they represent a gaseous fuel that can be utilized in different power generation systems. The amounts and concentrations of the different gases resulting from the cogasification process will be dependent on the operational conditions of gasification process taking place in the bed and on the proportion of crude glycerol in solid waste mix that has been admitted to the gasification bed [27, 28].

The literature findings show that H_2_ and CO are main constituents of the produced syngas out of the gasification of waste fuel materials such as the ones used in this study (see [27, 28], e.g.). However, the final concentrations of these two gases at the furnace exit will in our case also be dependent on the extent of oxidation reactions of the produced gases that take place in the furnace above the gasification bed. The supplied air flow rates will play a major role in this regard. We conducted different experimental runs in the built gasification and combustion furnace under different air flow conditions. Overall air/fuel ratio was varied from as low as 2.7 to about 8.7. These limits represent air-deficient to intermediate air levels conditions. The lower limit was selected in such a way just to maintain sufficient heat release in the bed to sustain gasification in the bed, without giving the chance for much more oxidation of the produced syngas.

On the other hand, the intermediate limit of air/fuel gives more chance of oxidation reactions of combustible gases. This gives the chance to reflect more on the thermal energy qualities of the produced syngas. However, supplying more air beyond these limits was not adopted in this study as it would cause some aerodynamic and flame stability problems due to difficulty in maintaining such a solid fuel in the form used inside the fuel bed under high air flow conditions.

The water jacket surrounding the furnace allows for quantifying the heat released by the combusting gases above the bed and the differences in this heat between the different runs conducted; in the absence of instrumentation for the gas concentration measurements in this study may somewhat still give some insight into the variations of the gases produced by the gasification process.

To boost our understanding further of the pyrolysis and gasification of the waste material used in this study, thermogravimetric analysis (TGA) tests were conducted under air and nitrogen environments for dry green tree leaves alone and another time for the mix of crushed tree leaves mixed with crude glycerol with 25 percent on mass basis for the later constituent.

## 2. Experimental

In this research, experimental work has been conducted in two main directions. In the first a thermogravimetric analysis (TGA) of the used fuels has been conducted to assess the thermal degradation characteristics of the solid fuels used. In the second part of the experiments, the fuels used were tested in a lab-scale furnace setup to evaluate their performance as fuel for continuous combustion systems.

### 2.1. TGA Experiments

Thermal gravimetric analysis (TGA) has been carried out for the crude glycerol and for the dry tree leaves after crushing them into small flakes. Thermogravimetrical analyzer is an instrument that simply measures weight change during heating of a sample of the material to be tested versus temperature. It is a very effective technique to study chemical and physical phenomena as a function of temperature. The TGA analyzer model used is TGA 7 (Perkin-Elmer USA). A mass of 20 mg of the solid fuel was heated under nitrogen (or air) flow (100 mL/min) from 30 to 800°C using a constant heating rate of 5°C/min. The weight of the sample is then recorded at different temperatures. In what follows, the TGA results obtained are discussed.

### 2.2. Experimental Combustion Test Rig

Below is a description of the furnace setup used in the second part. Figure 2 shows a schematic of the furnace used in the experiments. It is a vertical cylinder. The furnace has a conical solid fuel bed located at its bottom. The fuel is supplied to the bed from one side of the furnace, as indicated in Figure 2. Combustion air is admitted to the furnace through the bottom base of the bed. A cooling water jacket surrounds the furnace; the jacket is insulated on the outside to minimize heat loss to the surroundings. External heating LPG system is added to the system to initiate the gasification and combustion at the beginning for only a few minutes, before self-sustaining gasification and combustion system is attained (see Figure 2).

In the initial starting period of the combustion process, the hot LPG combustion products are guided to penetrate the bed from the bottom. After a certain period of time (typically 2 to 3 minutes) has elapsed and after observing effective gasification of the solid fuel in the bed, LPG supply is stopped and only air with the desired amount is allowed to penetrate through the bed. Effective gasification was judged by the high temperatures measurements on top of the bed and the exhaust gases. These high temperatures reflect high levels of gasification and subsequent combustion of the generated syngas to the extent that reflects the ability to attain self-sustaining continuous gasification process, without the need for further support by hot gases coming from LPG combustion. A J-type thermocouple, with an accuracy of ±1.5°C, has been used to measure the temperature at the exit of the cooling water jacket. N-type thermocouples with an accuracy of ±2.5°C were employed to measure the temperatures at the top of the bed (in the middle of the upper cross section of the bed), as well as in the middle of the exit cross section of the furnace. The exit temperature of the hot water and the combustion gas temperatures were all recorded via a data acquisition system using the LabView software. For all runs, the mass flow rate of the cooling water was measured by a flow meter of the rotameter type with an accuracy of ±2% being used. Flow rates of combustion air were measured by a rotameter flow meter and a sensitive scale was used to measure the mass of solid fuel supplied. The maximum errors in the mass flow rate of air and fuel are 4% and 0.05%, respectively.

## 3. Results and Discussions

### 3.1. TGA Results under Inert N_2_ Pyrolysis Conditions

**Figure 3:**
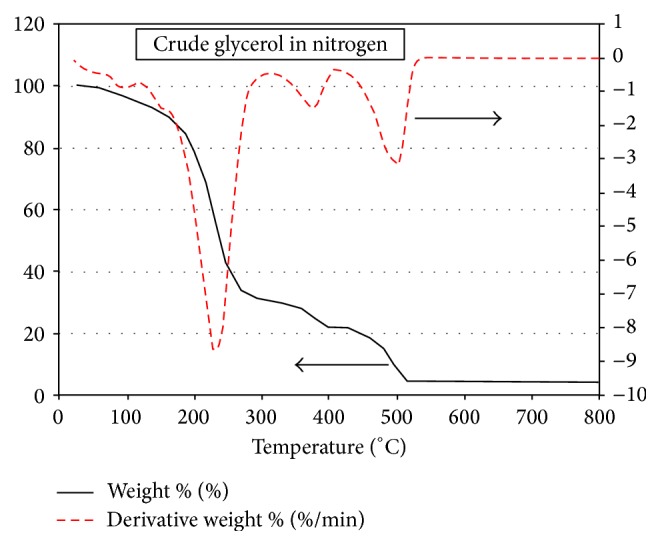
TGA and DTG curves for the crude glycerol in N_2_.

**Figure 4 fig4:**
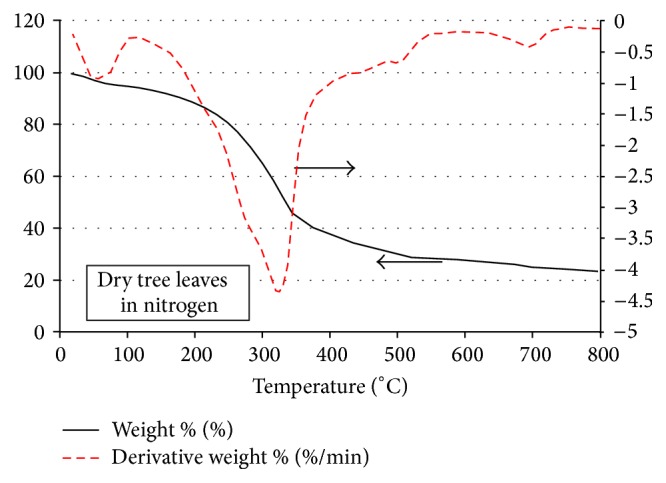
TGA and DTG curves for the dry tree leaves flakes in N_2_ environment.

**Figure 5 fig5:**
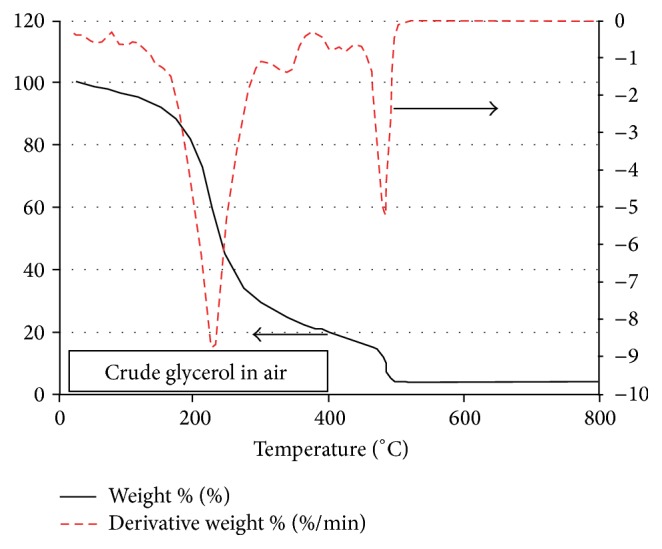
TGA and DTG curves for the crude glycerol in air environment.

**Figure 6:**
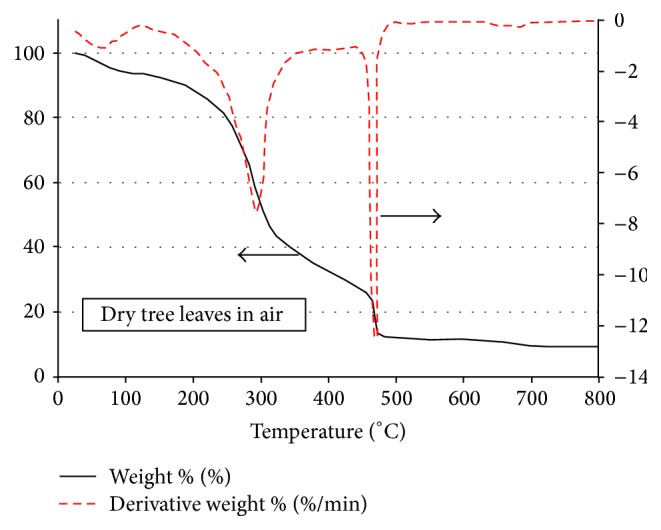
TGA and DTG curves for the tree leaves in air environment.

The thermal degradation results for the dry tree leaves flakes and the crude glycerol in nitrogen environment are presented hereby in the form of the TGA results and the corresponding derivative thermal analysis (DTG) curves shown in Figures 3 [Fig fig4] [Fig fig5]–6. The collected data are obtained in the range of temperature from initial room temperature up to about 800°C with a heating rate of 20°C/min. For both kinds of samples, the mass loss data show four phases of pyrolysis and gasification; the same observation was made in [26] in nitrogen environment. The temperature range of these four phases is given as follows:

 Phase 1: up to 160°C.
 Phase 2: 160°C to about 260°C.
 Phase 3: 260°C to about 515°C.
 Phase 4: 515°C to 800°C.

Figure 3 presents the TGA and the DTG curves for crude glycerol. The TG and the DTG curves show that the first phase starts to become noticeable at about 50°C and reaches a max mass loss rate at about 95°C and comes to an end at about 160°C where the second major phase that is associated with very fast mass drop starts. The first phase is associated with the removal of moisture and low temperature volatiles that both amount to about 10 percent of the original mass, while the second phase involves the devolatilization stage in which the major mass loss occurs mounting to about 54 percent and spanning over a temperature range from 160 to about 260°C.

Phase 3 spans over the temperature range from about 260°C to 515°C in which the total mass loss is about 31 percent that represents the degradation of fatty acids methyl esters and their residues resulting from the previous phase 2. The last fourth phase does not show any noticeable reduction in the mass up to a temperature of about 800°C indicating the presence of about 5 percent of ash and nongasifiable components by that temperature.

The thermogravimetric degradation curves for the case of only-dry-green leaves in nitrogen are shown in Figure 4. Unlike the case crude glycerol, in this case only simple smooth pyrolysis and gasification scheme is observed without being able to identify the four distinct phases observed in Figure 3 for the case of crude glycerol pyrolysis and gasification. Here, after the initial moisture removal phase the subsequent distinct three phases observed in Figure 3 seem to be merged and combined in a one simple phase (cf. Figure 4). The total mass loss by the time the temperature 800°C is reached amounts to about 77 percent, as compared with about 96 percent total mass loss in the case of the crude glycerol (Figure 3). The higher degradation in the crude glycerol case in phase 3 might be due to the possible catalytic effect of methanol and water present in the crude glycerol [26]. Moreover, in Figure 4 for the tree leaves case only one major negative peak is seen in the DTG curve during the major devolatilization stage while in Figure 3 two such major peaks are obtained, one in phase 2 and the other towards the end of phase 3.

### 3.2. TGA Results under Air Environment Conditions

Figure 5 presents the TGA and DTG curves for the case of crude glycerol but under air environment conditions. The four phases observed in the case of the pyrolysis and gasification of crude glycerol under nitrogen environment are also observed here under air environment.

Figure 5 shows, both quantitatively and qualitatively, the same four phases observed under both nitrogen and air pyrolysis conditions (see Figure 3) except that the air environment results in a backward shift by about 15°C for the second negative DTG peak observed towards the end of phase 2 (cf. Figures 3 and 5). Moreover, the char and ash remains in phase 4 at the temperature 800 are slightly less in this case under air environment than what has been observed under nitrogen environment in Figure 3.

The TGA and DTG curves for the tree leaves under air environment conditions are shown in Figure 6. Interestingly, the phases observed in the cases with crude glycerol are seen qualitatively to a good extent in this case for the tree leaves under air conditions, unlike the case of Figure 4 of tree leaves pyrolysis under nitrogen conditions. Quantitatively as well as qualitatively, phase 1 for this case is the same as that for the tree leaves case under nitrogen conditions. Phases 2 and 3 in this case, as can be seen from Figure 6, are merged in one single stage with a total mass loss at the end of which amounting to about 70 percent, which is the same amount for both phases 2 and 3 in Figures 3 and 5. One more interesting point about Figure 6 is the double peaks of the DTG curve and their absolute magnitude, where contrary to the earlier cases for the crude glycerol pyrolysis that were associated with double peaks on the DTG curves in Figure 6 the first peak is clearly smaller than its counterparts in Figures 3 and 5 and this peak takes place in Figure 6 at a clearly higher temperature (around 300°C) than those in Figures 3 and 5. The second peak of the DTG curve in Figure 6, on the other hand, is by far bigger than the first peak and is clearly bigger than the second peak levels of Figures 3 and 5. In addition to that, the presence of air leads to shifting the first peak location backward by about 25°C as compared with the case of tree leaves under nitrogen environment conditions (cf. Figures 4 and 6). At the temperature of 800°C, the total mass loss in Figure 6 is about 80 percent which is clearly more than the amount of mass loss in the case of tree leaves pyrolysis under nitrogen environment (cf. Figure 4).

## 4. Furnace Combustion Tests Results

To complement the discussion we presented above about the tree leaves and crude glycerol thermal degradation characteristics and to explore a suggested way to facilitate their use as fuels in furnaces, we used the furnace shown in Figure 2 to directly burn tree leaves and crude glycerol under different air flow conditions. Following the literature (see, e.g., [27–29] and the references cited therein), crude glycerol is mixed in this work with the crushed dry tree leaves with a mass percentage for the glycerol of 20 percent, targeting thereby enhanced gasification and production of clean and valuable syngas products (H_2_ and CO).

The furnace depicted in Figure 2 was used to conduct four main experimental tests. The first test was conducted with dry crushed tree leaves in the form of flakes while the other three tests were all conducted using the tree leaves flakes after being mixed and wetted with crude glycerol in order to give them more mass and energy density and tenacity that would expand the margin of their use under higher combustion air flow conditions. Due to the tree leaves flakes lightness, if they were not wetted with crude glycerol, it would be difficult to get them settling in the bed and spending effectively the required residence time in the gasification bed and subsequent combustion zone of the furnace where it will be easy for the combustion air and the gases flowing in the furnace to carry them and disperse them in uncontrolled manner that may adversely affect the gasification and hence the overall combustion process.

In Test 1 we supplied dry tree leave flakes to the fixed bed located at the bottom of the furnace (see Figure 2), with overall air/fuel ratio of about 6. In the other three tests conducted (Tests 2, 3, and 4) with glycerol-wetted tree leaves flakes as the fuel, the air/fuel ratio was varied from 2.7 to about 8.7. Details of the conditions of the different tests are given in Table 1. For all tests, the processes in the furnace were monitored over time and data were collected by a data acquisition system after having reached a consistent steady fuel feeding conditions at the desired air flow condition of any of the conducted tests.

Cooling water flow rate and the fuel feed rate for all tests are 30 g/s and 20 g/min, respectively. For all tests the inlet water temperature to the water jacket surrounding the furnace is about 23°C.

We start the discussion of the results with the heat transferred to the furnace walls. The amount of heat transfer from the flame and hot gases to the furnace walls, which is transferred by both radiation and convection during the tests, is assessed by measuring the amount of heat captured by the water jacket surrounding the furnace (see Figure 2). For that purpose, typical energy conservation analysis was applied to the water jacket.

**Figure 7:**
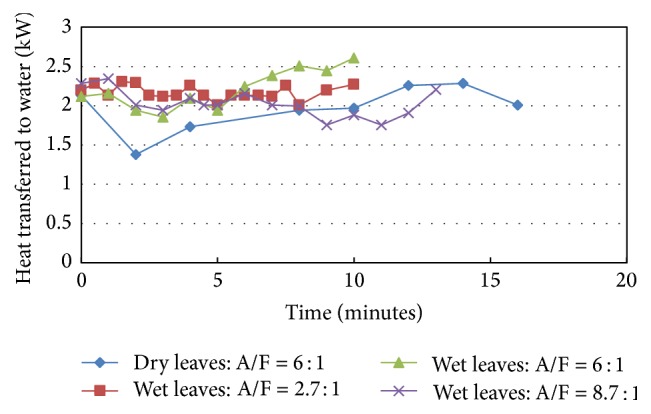
Heat transferred to water for dry and wet leaves.

Figure 7 shows the instantaneous results for heat transfer to the water jacket for the four tests. As can be seen from Figure 7, the wetted leaves tests results of Tests 2, 3, and 4 show both qualitative and to a good extent quantitative agreement among each other. On the other hand, the test with dry leaves (Test 1) shows a clear quantitative deviation from the other three tests where it showed less heat transferred to the water jacket. Figure 7 shows also more combustion stability and more stable heat transfer fluctuations with less amplitude for the tests with wetted leaves (Tests 2, 3, and 4) as compared with the results of the case of dry leaves (Test 1). For all tests with wetted leaves, more or less, the levels and amplitude of fluctuations in heat transfer results are almost the same reflecting almost similar combustion and performance dynamics for these cases and that the processes taking place in these tests are not very sensitive to air flow variations within the range of air flow rates addressed in these tests. On the other hand, dry air test is expected to be more amenable to changes in air flow rates where the light density of dry leaves is believed to lead to more transient behavior with time under different combustion and air or fuel feed conditions.

**Figure 8:**
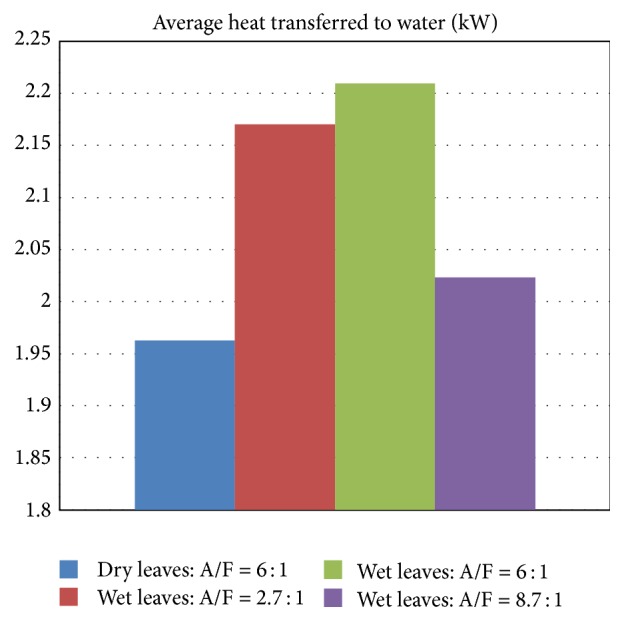
Average heat transferred to water for dry and glycerol-wetted leaves.

Average values of heat transferred to the water jacket during the test period are reported for the four tests in Figure 8. Test 1 with dry leaves shows the least level of heat transfer to the furnace walls among all tests conducted. By comparing Test 1 with Test 3, where both are under same air flow condition with air/fuel of 6, it is found that average heat transfer to the wall in Test 1 with dry leaves is less than that in Test 3 with witted leaves by about 10 percent. This difference between the two cases is attributed mainly to the expected differences, due to the presence of crude glycerol, in radiation from the hot combustion gases to the wall that resulted from the pyrolysis and gasification of the wetted tree leaves, which are expected to be different from those resulting from the gasification and combustion of the dry tree leaves. The heating value of the tree leaves (whether dry or wetted with 25 percent of crude glycerol) is not expected to play any significant role in this regard since they are expected to be very comparable to each other in both cases. Even to the contrary, when crude glycerol is mixed with tree leaves, the heating value (although not measured in this work, but argument is based on work reported in [27] for wood mixed with crude glycerol) is expected to be slightly lowered due to the presence of water in the crude glycerol. This argument leads us to believe that the main reason for the difference in heat transfer from the hot gases is the nature of the gaseous species present which are very much dependent on the gasification process and the raw fuel from which these gases emanated.

For Test 2 with lowest air/fuel ratio of about 2.7, the average heat transfer rate to the water jacket is about 2.17. Increasing the combustion air amounts further to reach air/fuel ratio around 6 in Test 3 (i.e., doubling the air flow rate) did not show any noticeable change in the results of heat transfer, with only about 2 percent increase (cf. Figure 8). However, by increasing in Test 4 the air amounts further to be about 3 times the value used in Test 2, a clear reduction in the thermal performance is noticed where a reduction in the rate of heat transfer to the water jacket by more than 5 percent is observed (cf. Test 2 and Test 4 results in Figure 8). This reduction is due to the cooling effect of the additional air supplied. In addition, given the light nature of the used material (tree leaves flakes) that easily can distort the operational stability of combustion and hence heat transfer and can pose difficulties supporting the flame based on such a light fuel under larger air flow conditions, there is still need for refinements in the design of combustion system to allow more effective combustion under high air flow conditions. One way to achieve this was the technique adopted in this work by wetting the light leaves flakes and thereby giving them more density.

One further interesting point to consider in regard to the above, in the conducted tests the bed and combustion taking place in the furnace take place at relatively large air flow rates that can be far above the levels needed for effective gasification that can lead to large concentrations of the valuable syngas components, particularly H_2_ and CO. In a modified version of the furnace used in this work, splitting the zones for gasification and for subsequent combustion of the resulting gaseous fuels, by using primary and secondary compartments, may lead to a better gasification and hence better utilization of the biofuels used in this work.

It is anticipated that crude glycerol would contribute to enhancing the radiating characteristics due to the presence of liquid and other gaseous fuel products that have possible enhanced sooting features when burned, over those of the products from the gasification of only dry tree leaves. It should be recalled that radiation is a main mode of heat transfer in combusting gases and it is enhanced by the formation of particulate matter in the hottest fuel-rich sites of the flame. From the obtained results it seems that glycerol supports and enhances the formation of soot particles in the combustion domain which ultimately enhances heat transfer by radiation. As such, wetting the tree leaves with crude glycerol, where both materials are considered to be a kind of biowaste, and subsequently using them for the purpose of generating power or heat, has good potential to contribute to renewable energy sources for systems where enhanced radiative heat transfer might be sought. Hence wetting the tree leaves with such a waste material like glycerol can be considered as an energy management opportunity of good reward and value that gives more significance to these two kinds of waste materials when combined in a way or another than using each one of them separately as a source of energy.

The use of crude glycerol with tree leaves flakes at the same time facilitates the burning of the glycerol in such a simple-design furnace with ease and at the same time gives more stability for the burning of the light density tree leaves flakes by giving them more mass density and hence more stability that otherwise would pose challenges when admitting them to the bed and the combustion domain and when attempting to get a self-sustaining and a stable flame resulting from their combustion.

The above results and arguments and conclusions that may be drawn from them support conducting more detailed investigations towards quantifying aspects like particulates matter emissions and emissions of any other possible pollutants resulting from the combustion of such combined fuels as the ones used in this study.

**Figure 9:**
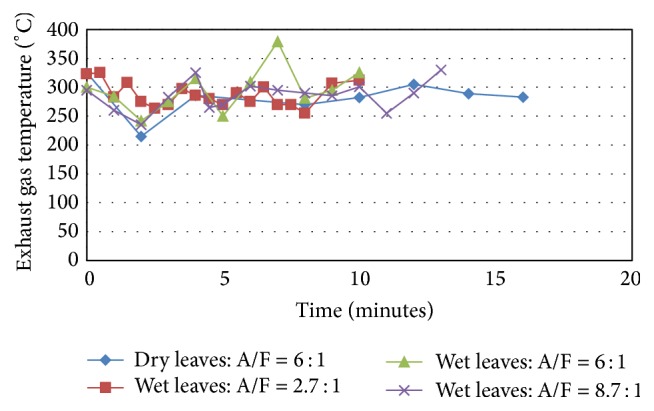
Exhaust gas temperature for dry and glycerol-wetted leaves.

The instantaneous dynamic behavior of some combustion results given in terms of average exhaust gas temperature over time, evaluated by measuring the exhaust gas temperature at representative points at the furnace exit, is shown in Figure 9. As can be seen, for all tests, the exhaust gas temperature, by virtue of the nature of the fuel feed process and the nature of the solid fuel flakes used, fluctuates and reported fluctuations are between a minimum of about 210°C and a maximum of about 350°C. Test 1 with dry leaves shows the least of these temperatures and the test with wetted flakes and air/flow rate scores highest among all tests. The reasons for the temperatures of Test 1 being lowest among all is not expected to be due to heating value issues of the used fuels as both fuels used have almost comparable heating value levels (as concluded from the work of other researchers on similar fuel combinations as the ones used here (see, e.g., [27])). The more possible reason however is the issues related to the controllability of the dry tree leaves flakes in the furnace due to their low density and their tendency to be easily dispersed randomly by the flowing gases in the bed and the other combustion zones of the furnace which will consequently affect the nature of the pyrolysis and gasification process taking place and then the resulting flame. On the other hand, more stable and stabilized fuel in the bed was attainable due to the stabilizing and the tenacity enhancement effect glycerol gave to the dry tree leaves flaxes, which contributed to attaining more stable combustion and hence higher exhaust gas temperature in the other tests.

Exhaust gas temperatures for Test 4 with wetted leaves shows lower values compared with Tests 2 and 3 due to the cooling effect of the extra air quantities supplied to the furnace in that Test. But also possible are effects on gasification and pyrolysis of the glycerol-leaves mix as the air supplied becomes more than a certain level. Many researches considered only the gasification of such glycerol-biomass solids mixes which normally take place at much more air-deficient conditions than the ones of the tests in this study. In these researches, the target was to get out syngas from the gasification process rather than supplying further air (as is the case in this work) to burn the resulting gaseous fuels and other possible tar and other liquid species out of the pyrolysis and gasification.

**Figure 10:**
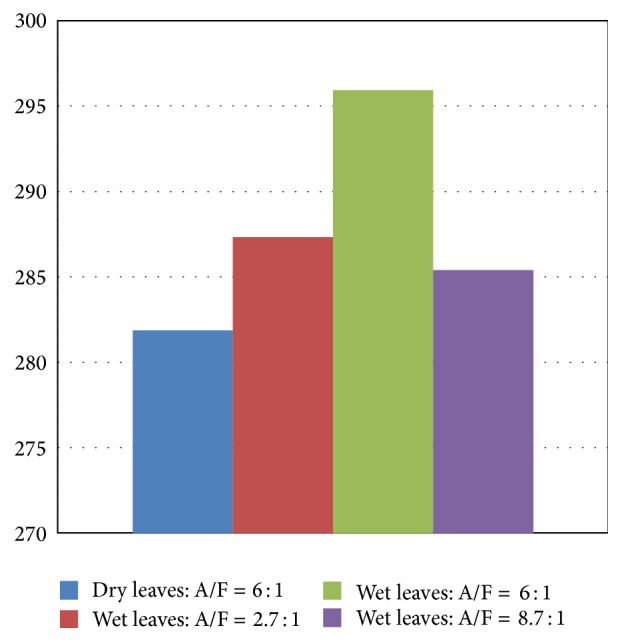
Average exhaust gas temperature for dry and glycerol-wetted leaves (°C).

Figure 10 shows the average exhaust gas temperature over all test time for all tests. The variations in the average temperature between the four tests is less pronounced, where the minimum recorded for Test 1 is about 282°C and the maximum corresponding to Test 3 is about 296°C.

**Figure 11:**
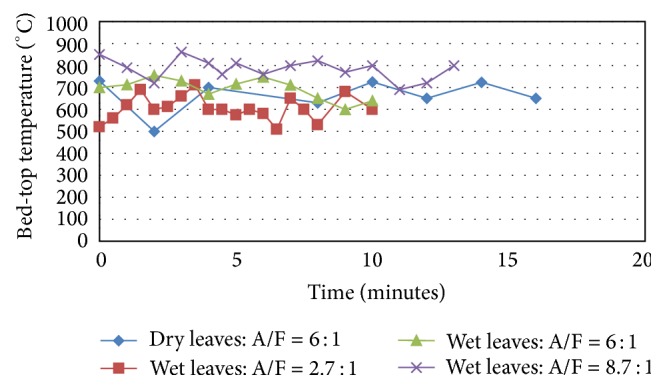
Bed-top temperature for dry and glycerol-wetted leaves (°C).

**Figure 12:**
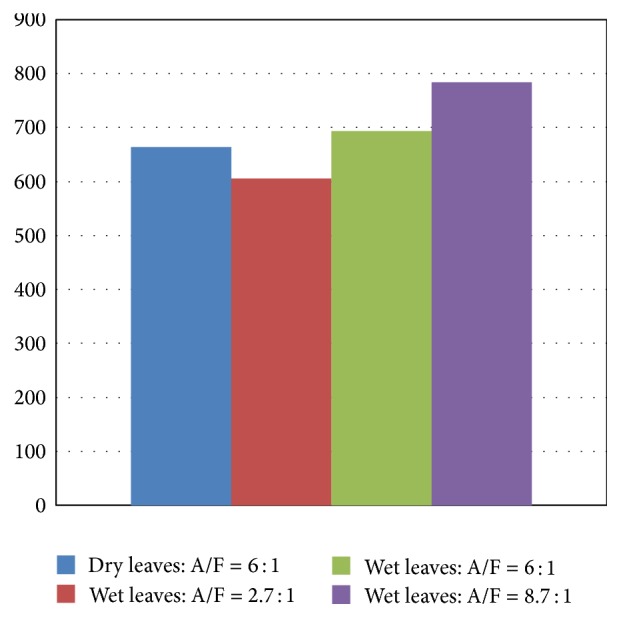
Average Bed-top temperature for dry and glycerol-wetted leaves (°C).

Finally, the gas phase temperature immediately at the center point on top of the bed is presented next in Figure 11. This quantity might be so helpful to get further insight into the kind of pyrolysis, gasification, and oxidation reactions taking place in the bed and also into the subsequent combustion taking place in the postbed combustion zones of the furnace. The average of the bed-top temperature for the different tests over time is presented in Figure 12.

Figures 11 and 12 show that the cases with larger air amounts result in higher temperatures at the top of the fuel bed and the lowest temperature of the bed-top is that of Test 2. Even Test 1 of dry air showed higher bed-top temperature than that of the wetted leaves in Test 2. More air flow would mean more turbulent intensity at the bed top and hence more combustion intensity in the main combustion zone above the bed and hence higher temperatures. Moreover, this leads also to more oxidation of the fixed carbon in the bed leading to higher temperatures which consequently leads to increased completion of carbon reaction to form CO_2_ and hence liberate heat that would aid further the gasification and devolatilization of the fuels in the bed in the pyrolysis zone. Of course, excessive air amounts on the other hand, especially with such low density solid fuel flakes (tree leaves), are difficult to stabilize in a fixed bed like the one used in the present furnace, which would lead to lower exhaust gas temperatures, as indicated in the previous discussions. The average bed-top temperatures for the four tests presented in Figure 12 confirm the instantaneous dynamic behavior observed in Figure 11. The highest temperature is around 780°C and corresponds to Test 4 with largest air flow while the lowest temperature corresponds to Test 2 with wetted leaves but with lowest air/flow ratio and it is about 600°C. With this being said, in Test 2 it is expected that the unburned syngas concentrations are highest due to the deficiency of air in this case.

## 5. Summary and Conclusions

Tree leaves crushed in the form of flakes were considered as fuel for furnaces after being mixed with crude glycerol with a mass percentage in the mix of 25 percent for the latter. This has twofold benefits: First it makes it easier to control stable and effective gasification under wider range of air flow conditions for the light density tree leaves by granting them more density upon mixing them with heavier crude glycerol that aids the tenacity of the whole mix. Secondly, the syngas components resulting from the gasification of the leaves-crude glycerol mix show more effective combustion characteristics in the used furnace and have more favorable radiating features for heat due to their more sooting ability, as compared with the combustible gaseous species resulting from the gasification of dry tree leaves alone. The above two features suggest an easy and effective method to manipulate the two waste materials, tree leaves and crude glycerol, in a way that at the same time contributes to environment-friendly renewable energy sources. The study also provided insight into the TGA curves of both fuels separately under air and nitrogen environment. For the crude glycerol, the TGA and DTG characteristics are both quantitatively and qualitatively similar, regardless of whether air or nitrogen are used. For the tree leaves on the other hand, TGA results under air are clearly different from those under nitrogen.

Below are more specific conclusions based on the furnace combustion tests conducted:

1. Over the range of air/fuel ratio considered, wetted leaves combustion shows more stability and consistency with less dependency on the range of combustion air supplied. On the other hand, dry leaves flakes were possible to burn under stable conditions only under low air flow environment due to their low density.
2. The heat transfer to the furnace walls is found to be highest under air/fuel ratio of about 6. Increasing the air supplied by 50 percent beyond that would lead to a reduction in heat transfer to the walls by about 5 percent. Reducing air flow by 50 percent (reduction of air/fuel from 6 to about 3) on the other hand did not lead to much reduction in the rate of heat transfer to the walls.
3. The temperature of the exhaust gas was higher for the tests with wetted leaves than in the case of dry leaves due to more stable combustion and to higher energy density in the case of the tree leaves when wetted with glycerol than the dry leaves. The stability of the combustion and the fact that only low air amounts could be supplied in the case of the dry air test also contribute to the lower exhaust gas temperatures, which would mean incomplete combustion and unburned fuel and particulate matter emissions in the case of dry air compared to in the other tests.
4. The glycerol is expected to have some pronounced effects on the radiation characteristics of the flames and hot gases in case of wetted leaves, which may explain the superiority to heat the water (mainly) by radiation from generated soot particles in the case of the tests with wetted leaves.

## Figures and Tables

**Table 1:** Summary of the main conditions of the furnace tests.

Test number	Description	Air flow rate L/min	Air/fuel
1	Only dry tree leaves flakes	100	6
2	Tree-leave flakes-crude glycerol mix	45	2.7
3	Tree-leave flakes-crude glycerol mix	145	8.7
4	Tree-leave flakes-crude glycerol mix	100	6

## Nomenclature

A/F: Air to fuel ratio
DTG: Derivative thermal analysis
TGA: Thermogravimetric analysis.
